# Schooling Children

**Published:** 1860-01

**Authors:** 


### SCHOOLING CHILDREN.

THE outrages and stupidities practised in modern education are not amazing, for a sensible man is prepared for any thing, and has no amazement; but they are mischievous in the extreme. Who expects a young girl to know any thing as it should be known? If there is such an individual, he ought to be sent to Barnum's Museum, and the price of admission advanced fifty per cent!

There are good schools here and there, but three out of four are the merest shams, are perfect impositions. Too much is attempted, hence much is passed over, but there is thoroughness in nothing. The young ladies know nothing well. We knew a graduate of one of the oldest schools in this city, make a mistake against herself of fifty per cent, in a bill against us for the private tuition of two of our children.

We believe the public schools in New-York, especially as to girls, are making an admirable change in this regard; we know more particularly of the Twentieth Street School, under the presidency of Miss Purdy, and of the Twelfth Street, of which Miss Greer is the principal. These ladies are remarkable for their energy, system, tact, and sound judgment, and merit well of the public. We trust they will long occupy their places and put the community under still higher obligation to them, for their fidelity to their trusts. Of the sub-teachers, the names which we have heard mentioned by different persons, with most praise for their assiduity, their conscientiousness and their unwearying patience towards the children under their care, are those of Misses Moran, Turnbull, Corneille, Thompson and Carpenter, connected, we believe with the schools named.

In all our public schools there is considerable need for amendment in several directions. We have before insisted on the wisdom and humanity of reducing school hours, to all under twelve years of age, to four a day; two in the forenoon and two in the afternoon; and that nothing whatever should be given to the children to learn, out of school hours. But this is so far ahead of this driving age, that we fear we shall be as gray as a rat and as blind as a beetle, before such desirable changes come.

There are several things which could be easily remedied, and doubtless would be, if they were properly brought before the teachers, superintendents, and trustees. The best way to do this would be to appoint us with a liberal salary, to make the circuit of the city schools once a month, the year round, and beat some common-sense into the craniums of those who need the commodity. It is of some importance to parents to have healthy children. Nothing can afford any solid satisfaction when a dear child is sick; for then, there is a cloud hung all over the world, and the brightest sun is veiled in black. Those who know most are the most alarmed at the slightest ailment of a child, for no one can conjecture what any sickness will end in. On the other hand, when every child is well, how the countenance brightens up; how the heart rises in its gratitude and its gladness, and how the whole world is changed!

To have a child go out to school in the morning in joyous health, and to come home with a broken limb, a gashed face, a lost tooth or an endangered eye; or to be waked up in the night by the ominous sound of the dreaded croup, or a putrid sore throat, or the more insidious scarlet fever! Any one of these things, by their suddenness, is well calculated to send terror into a parent's heart. All of them may be said to be of daily occurrence, and yet all of them are more or less avoidable.

A month or more ago, we heard a little girl of ten, complaining in the street of her hard lesson. On inquiry, we found that between four o'clock of a winter's day and the hour of school next morning, her teacher had required her to get the meaning of all the words, which she thought she did not know —in a hundred pages, twelvemo; and besides this, there were two other lessons. One might well suppose that such a teacher had been lately imported from a lunatic asylum.

One of the down-town schools on the late Thanksgiving occasion, when it was desired to dismiss school from Wednesday night to Monday morning, the teacher gave out lessons for three ordinary days, on the ground that the children had a long holiday. We did not inquire, nor do we know the teacher's name, but no doubt it will become famous one of these days.

Within three months a public examination took place in one of the schools, a class at a time. When one was under examination, requiring three quarters of an hour, another, numbering perhaps fifty, from eight years to thirteen, were told that they must steadily look at a certain spot on the wall during the examination, and that whoever turned the head, or was restless, should be "kept in" after the school was dismissed at three o'clock, for every afternoon during the remainder of the week; one little girl is reported to have grown sick, and perhaps fainted away, under the ordeal. We do not know that this is literally true, not having seen it; but such is the report of "visitors," on the occasion referred to. If the report is pretty nearly correct, the teacher who gave the order merits the severest reproof.

Partially informed persons have an over-dread of foul air. We know a teacher, who, during winter, has a company of several little girls in a room, but fearing the effects of breathing the air over and over again, where the heat comes from a register, she keeps the sash down several inches near the ceiling, for purposes of ventilation; but the air rushes in with great power on a winter's day, and drives directly upon the heads of the children in a steady cold stream, and they sitting still, must experience disastrous results, such as cold in the head, sore throat, fevers and croups. A wiser plan would be to allow the children to promenade the hall for ten minutes every hour, and during that time open the window to its utmost and the door also, thus causing a most thorough ventilation. No person can sit still in a warm room in winter in a draft of air for five minutes without injury; but for children to be thus exposed by the hour is monstrous. As the feelings are very deceptive, there should be a thermometer in every school-room, at about five feet from the floor, and at the coldest part of the room, where it should never be allowed to fall below sixty, nor to rise higher than sixty-five.

With a view to obviate the hurtful effects of confinement, some of the public schools give a few minutes every hour, for the children to recreate. Usually, they are sent down into the yard, and it is forbidden for any child to stand still. This is a most judicious arrangement in the main. But if the thermometer is at thirty, it is freezing cold out of doors, and even if twenty degrees higher, if there is a raw wind blowing, which makes it equivalent to thirty, the change from the school-room is not less than near forty degrees, and nothing short of very active running or play can avert bad colds, croup, pneumonia or pleurisy. It would be far better, because entirely safe, to make the children exercise in the hall of the building, when the thermometer was under thirty-five, especially if a cold wind was blowing.

It ought to be remembered by all, that it is far safer and much less disastrous to breathe any ordinary bad air, if warm, than to be in the purest air on the globe, if it is cold enough to cause a general chilliness, or a partial feeling of cold for a very short time, such as on the back, or neck, or throat, or any other susceptible part. Children should not be allowed to sit for five minutes with their backs to a register, or stove, or fire; nor to stand over registers for a moment, nor to sit near one for any length of time; and in cold weather they should be made to bundle up before leaving the school-room, and be counselled to run home and not delay a single moment on the way.

